# Liberal/Individualized Versus Materialist/Structuralist Approaches to Addressing Social and Health Inequalities: Education and Income as Social Determinants of Health

**DOI:** 10.1177/2752535X251316086

**Published:** 2025-01-26

**Authors:** Avery Ervin, Dennis Raphael

**Affiliations:** 1 School of Health Policy and Management, 56014 York University, Toronto, ON, Canada

**Keywords:** education, income, social determinants of health, health inequalities

## Abstract

**Background: **While consensus exists that the sources of health inequalities are social inequalities brought on by the experience of qualitatively different living and working conditions, means of addressing these conditions continue to be the subject of dispute. Whether to emphasis education or income as a social determinant of health is one such example of differing views on the sources of these inequalities and the means of addressing them. These different emphases are often justified through the narrow examination of the magnitude of statistical relationships between educational attainment and income with health outcomes.

**Purpose: **We offer a broader view, seeing these differing emphases as indicative of contrasting views of the nature of society and means of responding to these inequalities with emphasis on education representing a liberal reformist view of the issue while an emphasis on income representing a materialist structuralist view.

**Research design and study sample: **We examine, the validity of this hypothesis through an analysis of content of five representative publications that consider educational attainment as a social determinant of health and five that do so for income.

**Analysis and results: **We find that the emphasis on education as a social determinant of health focuses on the attributes of the individual and is generally accepting of the structures and processes of the existing economic and political order. In contrast, an emphasis on income – when placed within a materialist analysis – views existing systems as inequitably distributing income and other resources thereby requiring their reform or transformation.

**Conclusion: **Considering evidence of deteriorating living and working conditions for many in Canada and elsewhere, we see the latter emphasis as more useful for understanding and addressing these disturbing developments.

## Introduction

Despite the explosive growth of knowledge concerning the social determinants of health and their role in creating health inequalities, there is little evidence of their being taken seriously by governing authorities and policymakers in many jurisdictions and this is especially the case in Anglo-Saxon liberal welfare states such as Canada, UK, and USA.^1^ This is due in part to the existence of competing discourses or explanations of what social determinants of health are, how they shape health, and the appropriate responses to their problematic quality and distributions among the population.

Raphael^2^ and Raphael and Bryant^3^ identified a variety of competing discourses which consider the social determinants of health. These include social determinants identifying those who require health and social services, how public policy shapes their quality and distribution, and considering their quality and distributions result from the structures and processes of capitalist economies.

In this article we focus upon another issue that hinders addressing the social determinants of health: whether emphasis should be placed on education or income as a social determinant of health.^1^ We believe these views are built upon differing paradigmatic assumptions concerning the nature of society, the structures and processes that create the social inequalities that drive health inequalities, and the appropriate means of improving the quality and equitable distribution of the social determinants of health. We argue that emphasizing education as a social determinant of health rests upon liberal/individualist notions of the nature of society and societal change, and effectively casts the issue as one of remediating individuals’ deficiencies. In contrast, emphasizing income within a materialist/structural perspective directs attention to how features of economic and political systems inequitably distribute resources and the necessity for reforming or transforming them. These two approaches overlap in some conceptualizations and applications, but for the most part they do not, representing different understandings of the source of social and health inequalities and means of addressing them.

We link the education versus income emphasis to a broad critique of contemporary liberal democracy and its embrace of the capitalist market economy as the preferred economic system. Education and income approaches are usually also embedded within research traditions of positivism – concern with the concrete and observable -- versus realism -- concern with uncovering the societal structures that create these observables.^4^ We also relate these differing emphases to Levitas’s social exclusion discourses of MUD (Moral Underclass), whereby those experiencing adverse circumstances are seen as outside the cultural mainstream; SID (Social Integrationist), where difficulties are due to individuals’ failures to participate in the labour market; and Red (Redistributive), where adverse circumstances result from the structures and processes of an unjust society.^5^

We provide illustrative examples of how the education versus income emphasis plays out in research, practice and policy recommendations. We then show how the Canadian Pathways to Education project.^6^ – the most developed and known in Canada -- that aims to prevent high school leaving fits within the education approach and comment on how it has come to be directed by the Canadian corporate sector whose employment practices, anti-union activities, and advocacy for a reduced welfare state make such programs necessary. While such education programs have tangible benefits for some, they obscure and divert attention from the more important drivers of social and health inequalities: the structures and processes of economic and political systems that inequitably distribute power and influence, income, and other social determinants of health.

## Background

### Health Inequalities, Social Inequalities, and the Social Determinants of Health

Health inequalities are differences in health outcomes and access to healthcare among individuals occupying different social locations in society.^7,8^ Since most of these health outcomes and access to health differences are avoidable, the term health inequities is often used to make explicit that these differences are unfair and unjust.^8^ The source of health inequalities are social inequalities.

Social inequalities are differences in life circumstances and have been described in different ways.^9^ Sociologists and societally-oriented health researchers and workers usually direct attention to the structures and processes of societies that create these differences by placing people into differing social locations of relative power and influence that either provide or limit access to resources. Psychologists and individually-oriented health researchers and workers usually focus on individuals’ attributes avoiding the concept of social location and existing social relations between these locations in favor of descriptive terms such as socioeconomic status, occupational status, educational attainment and income group.

While conceptually, terms such as socioeconomic status, occupational status, and income clearly imply differences in power, influence and access to resources, it has been noted that these are primarily static descriptive terms rather than indicators of social relations, a point made explicit by those employing the concept of social locations.^10^ We see these differing concepts as indicators of differing paradigmatic approaches towards understanding and responding to phenomena such as health inequalities.

Grabb’s definition of social inequality leaves room for the importance of individuals’ attributes such as educational attainment and income – while Crossman’s definition directs explicit attention to the structures and processes of society^9,10^:

Social inequality can refer to any of the differences between people (or the socially defined positions they occupy) that are consequential for the lives they lead, most particularly for the rights or opportunities they exercise and the rewards or privileges they enjoy.^9, p.1^

Social inequality is characterized by the existence of unequal opportunities and rewards for different social positions or statuses within a group or society. It contains structured and recurrent patterns of unequal distributions of goods, wealth, opportunities, rewards, and punishments.^10^

Sociological expositions of the sources of social inequalities such as Grabb’s^9^ identify how “structures of domination” control resources through economic processes of extraction, production, finance, and commerce and political processes by the executive, judiciary, civil service, police and military. These structures also include the religious, media, educational, and scientific institutions that justify such domination by controlling knowledge. Not surprisingly then, those concerned with social inequalities usually direct their attention to how the distribution of income and wealth – as well as educational attainment – are determined by societal structures rather than the educational attainment of the individual.

Since health inequalities result from social inequalities, the components of social inequalities are also social determinants of health. The World Health Organization defines these as the conditions in which people live, learn, work, and play.^11^ Most conceptualizations include early life, education, working conditions, food security, health services, housing, and income and its distribution. By 2015, there were at least 36 such frameworks – more have since been generated – all of which include education and income.^12^

The social determinants of health concept developed out of a concern with how political and economic structures and processes impact health.^13^ Over time the concept became operationalized as a focus on health inequalities between groups characterized as occupying social positions of differing power, influence, and access to resources. Graham distinguishes between social determinants of health – for example, education and income are predictors of health -- and social determinants of health inequalities – for example, the inequitable distribution of education and income create health inequalities -- with the latter explicitly directing attention to the structures and processes of society that inequitably distribute health-related resources.^14^ We focus on health inequalities and their determinants in this article.

Education and income are found in virtually all models of the social determinants of health and the social determinants of health inequalities such that they occupy prominent positions in theory and research on health inequalities.^12^ Discussions of the quality and equitable distribution of both education and income can be concerned with the structures and processes of society, such as how certain groups based on class, gender, and race are excluded from access to education and income. Focus can also be on education and income as characteristics of the individual with rather less attention directed to the aspects of societies that create their inequitable distributions.

There is no reason that those promoting health equity cannot be simultaneously concerned with both education and income as social determinants of health. In our experience however, we have found that in practice, there tends to be two camps, one primarily concerned with education, and one primarily concerned with income as a social determinant of health. We believe that those concerned with education are usually focused on the attributes of individuals operationalized as educational attainment and psychosocial processes such as information processing, decision-making and potential for occupational advantage. The way it plays out is as a depoliticized and individualized focus on educational attainment directing attention away from *Who Gets What*, *When*, *How?* to identifying the deficiencies of individuals.^15^ In contrast, we believe those concerned with income usually direct attention to how the structures and processes of society distribute income and other resources.

Among these different emphases -- education versus income as social determinants of health -- the paradigmatic views behind them are almost always implicit. We believe that in research and practice these emphases do not usually overlap since they are rooted in profoundly different orientation towards understanding societies, how these societies distribute resources, and the means of rectifying inequalities in these distributions.^2^ The main purpose of this article is to make these differences explicit and discuss their implications for promoting health equity.

### Education and Income as Social Determinants of Health: Making the Implicit Explicit

Education and income are viewed as important social determinants of health due to their strong statistical associations with health inequalities. Usually not discussed is how the emphases ascribed to them is a function of paradigmatic views held by their proponents of *ontology* – what is the nature of reality – and *epistemology --* how to go about learning about it. Wilson terms these research traditions,^4^ Lincoln and Guba paradigms,^16^ and Foucault,^17^ among others, discourses. In this paper we make these traditions, paradigms, and discourses explicit and detail how they inform understanding of the emphases upon educational attainment versus income dichotomy.

While research in both approaches may say little of how broader aspects of society shape their character, we believe this deficiency is much more common among educational attainment proponents. The tendency to narrow focus to the individual rather than structural aspects of society therefore lacks what C. Wright Mills called *The Sociological Imagination* as summarized by Ekta^18^:

The sociological imagination involves a shift from a personal or individualistic view of problems to a broader perspective that considers the larger social, economic, and political forces that shape individual experiences. It allows individuals to recognize that their personal troubles are often the result of broader social issues, such as inequality, poverty, discrimination, and structural barriers.^18, p.1^

Interestingly, we found little evidence of an explicit income versus education debate in the literature although those concerned with education usually argue for the value of the importance of education over income. Usually, these two camps tend to take little notice of each other. We believe this is because the camps represent different paradigmatic approaches towards understanding the sources of health inequalities and means of reducing them. The directing function of differing paradigms and discourses of knowledge was first popularized respectively by Kuhn^19^ and Foucault^17^ reinforced by the presence of differing discourses of the sources and means of reducing health inequalities,^3^ Indeed, this is why we argue an analysis of these approaches is necessary.

### Education as a Social Determinant of Health

The educational attainment viewpoint is buttressed by statistical analyses of education often being a better predictor than income of health outcomes, thereby implying it be given emphasis.^20^ The mediation pathways provided between educational attainment and health inequalities are occupational attainment leading to higher income, ability to process health-related information, and better understanding and ability to act upon one’s world (see below). Usually ignored are the societal structures and processes that make educational attainment such a potent predictor of income and health in some nations, but less so in others. The association is weaker where collective employment bargaining and union density that equalizes wages are higher, tax structures that redistribute income more progressive, and social spending that provides financial benefits and supports is more generous.^21,22^ Educational attainment adherents may call for improving educational systems, but attention is usually directed to individual-focused mentoring or tutoring.^6^

### Income as a Social Determinant of Health

The income viewpoint is also supported by statistical associations of income with health outcomes. However, these efforts usually note how income is also related to a slew of other social determinants of health such as food insecurity, housing unaffordability and unsuitability, insecure employment and poor working conditions, and unsafe neighborhoods across the income range. The pathways from income to health are experiences of material advantage or deprivation, psychosocial effects of such advantage or disadvantage, and adoption of coping mechanisms such as tobacco and excessive alcohol use. Income is especially important as a determinant of health inequalities because it has long been noted that market economies are organized such that income inequalities are built into its emphasis on capital accumulation – or profit making – with its most egregious outcome being the generation of poverty, a profound predictor of adverse health outcomes.^23^ As a result, work on income is more likely to consider how income and its distribution are related to societal structures and processes, a focus usually not seen in studies of educational attainment as a social determinant of health.

Since income is related to educational attainment – itself related to health outcomes – these associations provide a window for educational attainment adherents to argue that attaining education can either (a) avoid low income or (b) ameliorate the effects of low income upon health, thereby improving health without addressing inequitable distributions of income. The former assumption is questionable due to the extensive presence of low-waged labour and the phenomena of underemployment whereby attaining educational credential is no guarantee of employment that provides living wages. The latter assumption is also questionable as low income not only affects educational attainment but is associated with a range of health threatening living and working circumstances that remain unchanged during the process of educational attainment (if such attainment was possible): adverse housing conditions, food insecurity, stress of parents and children, and adoption of problematic coping mechanisms that threaten health.

### Conceptualizations Behind the Educational Attainment vs Income Debate

We see these differing conceptualizations as embedded within views of the nature of society that are usually not made explicit. At the broadest there is a dichotomy between a liberal/individualized worldview that focuses on identifying and acting upon individual attributes vs a materialist/structuralist worldview concerned with identifying and acting upon the societal structures and processes that produce and distribute resources.^24^

### Liberal Conceptions of Society

Liberalism/individualism as a political ideology has a complex history and is both lauded as a celebration of individuality and enterprise and condemned as justifying profoundly unjust societal arrangements. Freeden^25^ outlines five layers of liberalism which manifest differently in time and place: (1) liberalism as human rights unimpeded by government action; (2) liberalism as unimpeded market activity; (3) liberalism as unlocking human potential and encouraging individual development; (4) liberalism as protecting people from threats through communal action, including governmental and (5) liberalism as dispensing with the unitary view of society of level four and emphasizing the protection of minorities through an identity-based rather than class-based politics. The emphasis on individualism and the resurgence of layer two and weakening of level four apparent in contemporary neoliberal thought and governance is consistent with belief in educational attainment as a social determinant of health. Increasing concern with level five reinforces the focus upon the attributes of those in particular groups.

Macpherson^26^ argues in his analysis of liberal democracy – the system common across just about all western nations – that the key feature of liberalism and its manifestation in liberal democracy is individual freedom within the market economy with provision of economic and social security secondary. The educational attainment position is clearly compatible with this dominant liberal concept.

Macpherson’s concerns have been reaffirmed by the acceptance of many aspects of neoliberal governance across all forms of the modern welfare state.^27,28^ A focus on individuals’ attributes such as lower educational attainment and integrating them into the existing market economy is one of many health-promoting approaches that target individuals:

These approaches are aimed at developing individual characteristics within certain individuals or groups. They entail strategies aimed at improving knowledge, attitudes or behaviours such as education, literacy, physical activity, individual support, empowerment, the capacity to act, mindfulness, etc. Within these approaches, the absence of such characteristics is considered to be the cause of the deficiencies or disadvantages within certain groups, for example: limited personal knowledge, certain beliefs, low self-esteem, low levels of competence or lack of power.^29, p.7^

### Materialist Concepts of Society

In contrast, materialism – frequently manifested in calls for reform or transformation of the economic system – directs attention to the structures and processes of society and how the material basis of production and distribution shape people’s lives.^30^ Regarding income and its distribution, its most common exposition is in the form of critiques of current forms of macro-level public policy and/or the reforming or transformation of the political economy of a nation. For Mantoura and Morrison^29^ these two are respectively:

The approaches which focus on macrosocial policies tend to suggest ways of reducing inequality through broad social policies but do not necessarily question the ways in which the structures and ideologies of governance define the extent to which this is possible. These approaches tend to favor policies that provide the conditions concomitant with the underlying ideological structures of governance of the state (universal health care, in liberal democracies, for example, or provisions for daycare in social democracies). These universal policies are often seen from this perspective as best applied in combination with provisions targeting the most disadvantaged.^29, p. 6^

Political economy refers to a theory and an approach which, when applied to health inequalities, attempts to look at the assumptions and ideologies that underlie political and state structures and the effects that these have on populations. Political economy focuses on power and where it is concentrated in a society and examines how policies tend towards producing and maintaining inequality. Work on health inequalities from this perspective often emphasizes the need to fundamentally alter the nature of the role played by the state in liberal democracies so that it more closely resembles democratic states such as those found in Nordic and Scandinavian countries.^29, p. 6^

Clearly, focus on income and income distribution is more likely to fall within these materialist approaches than the educational attainment viewpoint. Also related to the liberal versus materialist view of society are research traditions that focus on measuring individuals’ attributes versus uncovering underlying societal structures that generate these observables.

### Research Traditions

Wilson^4^ provides a robust analysis of how differing research traditions in sociology – positivism and realism – direct attention to the characteristics of individuals versus the societal structures in which they are embedded. The research paradigm of positivism plays out as a concern with concrete observables usually focused on individuals and their attributes. It is noteworthy for its professed belief of objectivity and reluctance to engage in critical analysis of underlying structures and processes behind these observables. Emphasizing education as a social determinant of health is consistent with such a tradition.

In contrast, realism is concerned with the underlying and not readily apparent, structures of societies and institutions and the mechanisms that activate these structures to produce these concrete observables. It does not hesitate to make normative judgments as to preferred versions of society. Advocates emphasizing income as a social determinant of health are much more likely to work within this tradition.

We therefore see those emphasizing education as a social determinant of health usually conforming to positivist notions of reality: focused on the concrete and observable aspects of individuals’ educational attainments with rather little if anything to say about the structures and processes of society. Their analyses are usually quantitative, using the individual as the unit of measurement, and employing observable concrete variables such as educational attainment and health indicators.

In contrast, those emphasizing income are more likely concerned with uncovering the structures and processes of society that distribute income and other resources. Their focus is on how power and influence, economic and political structures, and the mechanisms which activate these structures, distribute income and other resources. While their analyses may also involve quantitative measures of observable variables such as income and health outcomes, there is attention given to making explicit the underlying structures driving the distribution of income and health outcomes and means reforming or transforming these structures.

### Changing Individuals vs Reforming or Transforming Society

Placing the locus of causality for health inequalities in educational attainment requires raising these levels. This can take place at an individual level through mentoring and tutoring or at a systems level by improving the overall educational experience through educational reform. We believe the emphasis is usually on the former, although in either case, these approaches direct attention away from the features of society that make education such a potent predictor of income: lack of labour laws and regulations that promote unionization, progressive income tax policy that redistributes income, and public policy that provides benefits and support through social spending.^31^ All of these would both raise the wages of those at the bottom of the income distribution and narrow the distribution of income, likely reducing the social inequalities that drive health inequalities.

Educational attainment adherents are usually unconcerned about problems of resource distribution related to the relative power and influence of the corporate and business sector versus organized labour. In a society where imbalances in power and influence are greater, there is a higher proportion of low-waged employment such that achieving higher education attainment for all – if that was even possible – would not remove the social inequalities – and resulting health inequalities – associated with low-waged labour.^3^ Simply calling for and possibly even achieving higher educational attainment will do nothing to influence these. These issues also play out in differing discourses of social exclusion.^5^

### Discourses of Social Exclusion

Levitas^5^ explores discourses of social exclusion with clear relevance to the educational attainment versus income issue. These are moral underclass (MUD), redistributionist (RED), and social integrationist (SID).

The MUD discourse views social exclusion – or in relation to our concern with individuals with lower educational attainment and/or income – as reflecting their motivational and moral failings. These individuals come to be so by virtue of their deviant habits, lifestyles, and attitudes. Related to this discourse and potentially related to the education versus income issue is the belief that generous welfare and program benefits create dependency, thereby reducing motivation to work. Limiting concern to raising educational attainment of those experiencing social and health inequalities opens the door to such interpretations. Even when individuals of low educational attainment are seen as victims of poorly designed educational systems or characteristics of society, the solution is to improve their educational attainment, not reform or transform aspects of the societies creating these conditions.

In contrast, the RED discourse views social exclusion – or in relation to our concern with individuals occupying low educational attainment and/or income – as resulting from the systematic exclusion of individuals from the economic and social resources required for participation in society. Social exclusion results from the failure of society to meet the economic and social needs of its citizens by providing meager benefits and low-paying and insecure employment and making the attainment of education difficult through high tuition costs. It also argues that one of the causes of social exclusion is unjust allocation of resources to the wealthy. The response should be producing public policy that provides wages, benefits, and assistance that allows people to live lives free of poverty.

The intermediate SID discourse also lends itself to an educational attainment viewpoint. The SID discourse sees social exclusion resulting from exclusion from the workforce. The usual direction involves bringing people into the employment market, with rights to resources through work or assistance de-emphasized. This is a key component of the educational attainment viewpoint: that educational attainment can provide access to better paying employment with little attention to the nature of employment.

The problem with this discourse is that for many, employment does not take them out of disadvantaged circumstances, and it says little about people unable to work because of illness, disability, or lack of employment opportunities. It ignores the structures and processes of society that inequitably distribute resources – including educational opportunity – among members of society.

### Implications of Differing Approaches and Discourses for Public Policy

Based on our familiarity with the literature we identified pathways by which educational attainment and income can be expected to be related to health (Tables 1 and 2). The analysis in later sections provides explicit examinations of these hypotheses.

**Table 1.** Four Primary Aspects of How Educational Attainment Comes to be a Social Determinant of Health.

Behavioural risk factors	Tobacco use, excessive alcohol consumption, lack of exercise, and poor food choices associated with lower educational attainment determines health.
Socioeconomic status	Educational attainment determines quality of employment – conditions, wages, and security – which determines health.
Access to healthcare	Those with less educational attainment lack health literacy and the skills to navigate the healthcare system, thereby determining health.
Personal cognitive and decision-making skills	Greater educational attainment provides skills that promote greater understanding and navigating the life course, thereby determining health.

**Table 2.** Five Primary Means of Conceptualizing How Income Comes to be a Social Determinant of Health.

Behavioural risk factors	Individuals’ smoking, drinking, exercise, and general lifestyle associated with income determines health.
Socioeconomic status	Elements of individuals’ environment, e.g. hazards at work, housing quality, employment or employment, and ability to purchase goods determine health.
Access to healthcare	Those of lower income lack health literacy and ability to navigate the healthcare system.
Structures of society	Societal structures (e.g., extent of redistribution, social spending, and nature of the workplace such as extent of unionization and collective agreements) shape the distribution of income and determine health.
Political economy	Also having a focus on the structures of society, the political economy perspective adds concern with how imbalances of power and influence within the capitalist economic system and its form of welfare state shape the distribution of income and determine health.

## Methodology

In this paper, we conduct a critical case study, informed by a critical social research perspective, of how education and income are conceptualized as social determinants of health. Harvey^32^ describes critical social research as situating social phenomena within the larger dominant social structures perpetuated and maintained through political and economic power and legitimated through ideological messaging. As discussed by Harvey, the case study researcher ‘deliberately selects, for detailed empirical analysis, a case that provides a specific focus for analysis of myth or contradiction’ (p. 153). In this case, how researchers and workers consider education and income as social determinants of health.

### Methods

Education and health have been extensively researched since the social determinants concept came into vogue in the early 1980s. For the period 1980-2000, Google Scholar returns 89 papers on “education and social determinants of health” and 50 on “income and social determinants of health.” However, from 2001 to the present, the returned papers are 17,500 on “education and social determinants of health” and 16,600 on “income and social determinants of health.” Rather than an exhaustive review of education and income-related articles on the social determinants of health, we identified five works that are not only good examples of emphasis on educational attainment^33-37^ and five on income^38-42^ as social determinants of health but also explicitly detailed the mechanisms that mediate the relationship between the social determinant of health and resulting health inequalities. Some are articles in peer-reviewed journals, one is a book chapter, one is a working paper, and one is a technical report from a foundation, but almost all have been influential as indicated by their number of citations. While some were written two decades ago – and this is especially the case for the income articles – their content continues to be widely cited as relevant. Readers can assess the validity/generalizability of our analysis to other works. The works examined for educational attainment and income are provided in Tables 3 and 4 respectively.

**Table 3.** Five Works on Educational Attainment as a Social Determinant of Health Examined and Number of Scholarly Citations.

Work	Citations
Low, M. D., Low, B. J., Baumler, E. R., & Huynh, P. T. (2005). Can education policy be health policy? Implications of research on the social determinants of health. *Journal of Health Politics*, *Policy and Law*, *30*(6), 1131-1162.	154
Cutler, D. M., & Lleras-Muney, A. (2006). *Education and Health*: *Evaluating Theories and Evidence*. National Poverty Center Working Paper Series# 06-19	2505
Hummer, R. A., & Hernandez, E. M. (2013). The effect of educational attainment on adult mortality in the United States. *Population Bulletin*, *68*(1), 1.	348
Hahn, R. A., & Truman, B. I. (2015). Education improves public health and promotes health equity. *International Journal of Health Services*, *45*(4), 657-678.	850
Zimmerman, E. B., Woolf, S. H., & Haley, A. (2015). Understanding the relationship between education and health: A review of the evidence and an examination of community perspectives (pp. 347-384). In *Population Health*: *Behavioral and Social Science Insights*. Agency for Health-care Research and Quality.	277

**Table 4.** Five Examined Works on Income as a Social Determinant of Health and Number of Scholarly Citations.

Benzeval, M., & Judge, K. (2001). Income and health: The time dimension. *Social Science & Medicine*, *52*(9), 1371-1390.	600
Marmot, M. (2002). The influence of income on health: Views of an epidemiologist. *Health Affairs*, *21*(2), 31-46.	1213
Coburn, D. (2004). Beyond the income inequality hypothesis: Class, neo-liberalism, and health inequalities. *Social Science & Medicine*, *58(1)*, 41-56.	837
Berkowitz, S. A. (2022). The logic of policies to address income‐related health inequity: A problem‐oriented approach. *The Milbank Quarterly*, *100*(2), 370.	8
Benzeval, M., Bond, L., Campbell, M., Egan, M. Lorenc, T., Petticrew, M. & Popham. F. (2014). *How does money influence health?* Technical Report. Joseph Roundtree Foundation.	134

Within these we examine the extent to which ideological and conceptual approaches to understanding and responding to the social determinants of health are present (i.e., liberal/individual vs materialist/structural understandings, individual vs societal focus, and affinities to the MUD, SID and RED discourses.

## Results

### Works on Educational Attainment

As noted earlier, we expected to see four main pathways by which educational attainment is seen as promoting health: behavioural risk factors, socioeconomic status, access to healthcare, and personal cognitive and decision-making skills.

Low, M. D., Low, B. J., Baumler, E. R., & Huynh, P. T. (2005). Can education policy be health policy? Implications of research on the social determinants of health. *Journal of Health Politics*, *Policy and Law*, *30*(6), 1131-1162.

This paper begins by citing sources that argue that income is an important social determinant of health but then states that since prospects for income redistribution are dim in the USA, a more palatable approach may be to promote educational attainment. Indeed, the authors quote Mark Penn, a pollster for Bill Clinton “People don’t want to see policies whose primary purpose is to redistribute income” and Democratic Senator Evan Bayh “We’re for distributing opportunities, not incomes.”

After detailing how both income and education are prime social determinants of health, the argument is then made that since education is seen as a non-partisan issue, promoting educational attainment offers the best opportunity for promoting health in the USA. This is especially important since there are wide gaps in educational attainment between various states, blacks and whites, and poor and other children. The striking rates of marginal or substandard literacy scores among Americans is another reason for such action.

The authors document the associations between educational attainment and health outcomes of self-reported health, levels of morbidity, mortality, and disability, health literacy, and health care costs. The pathways between educational attainment and health outcomes emphasize individual characteristics: “influencing work and economic conditions; enhancing social and psychological resources; enabling lifestyle and health behaviors; and directly, with no known mediator.” (p. 1139). Concerning the first pathway,

Well-educated people are more likely to work full time, have higher incomes, and be in more satisfying jobs. Better-educated people are less likely to experience financial hardship or to be unemployed (p. 1139).

The second pathway is educational attainment enhancing social and psychological resources such as marketable skills, “spiritual resources” for making moral choices, abilities to make informed judgments, increasing the sense of control, “enabling mastery,” and “facilitating self-direction.” Included in this pathway is educational attainment being related to “character development, self-control, self-efficacy, and resilience” (p. 1139). Finally, “Better-educated people tend to have more numerous, supportive, and informative associations with family, friends, and others in their community” (p. 1139) are factors identified as having positive effects on health.

The third proposed pathway is the well-worn positive health behaviours approach for people in general and especially for children, respectively:

Well educated people are more likely to engage in positive health behaviors such as exercising, not smoking, not drinking heavily, and using the healthcare system appropriately (p. 1140).

For children,

Academic success... influences future life choices. Its lack is strongly associated with youth problem or health-risk behaviors such as dropping out of school, tobacco cigarette smoking, using alcohol and other substances, violence, delinquency, risky sexual behavior, suicide ideation and behavior, unhealthy nutrition practices, and inadequate physical activity (p. 1140).

In addition to these arguments, the authors repeatedly emphasize that education is a better statistical predictor of health outcomes than income, an observation that while valid, does not certify that education should be the preferred focus of intervention for reasons cited earlier.

In one study examining the relative contributions of education, work, and income to health, Marilyn Winkleby and colleagues^43^ have shown that while income, education, and occupation all contribute to cardiovascular disease risk factors, the relationship is strongest for education (p. 1139).

Not only is education a strong predictor of health when occupation and income are adjusted, but a calculation of a direct effect of education (net of occupation and income) underestimates the total effect of education that (also) works indirectly by way of jobs and income,^44, p.729^, cited by authors (p. 1139).

The policy recommendations flowing from their arguments are that attempts should be made to carry out early intervention among American children in general and at-risk children in particular. The first reason is that these will effectively raise educational attainment across the nation in general, thereby improving health. The second, more problematic reason is that these interventions will somehow ameliorate the problems associated with profoundly unequal socioeconomic positions in the USA:

Because research shows such a strong association between education and good health, we offer evidence to show that at least two pressing problems in American society, namely the uneven distribution of educational attainment and health disparities linked to socioeconomic position, may be ameliorated through policy initiatives that link quality early childhood care, child development programs, and parental training in a seamless continuum with strengthened K–12 education (p. 1131).

The authors’ gaze is firmly placed on promoting individuals’ educational attainment as means of improving health. Identifying, much less addressing the USA’s economic and political structures that produce one of the highest levels of social inequality, are removed from consideration.

Cutler, D. M., & Lleras-Muney, A. (2006). *Education and health*: *Evaluating theories and evidence*. National Poverty Center Working Paper Series# 06-19.

This very well cited article illustrates all of the features associated with education as a social determinant of health approach. It is focused on individuals’ educational attainment with special attention paid to educational attainment levels’ association with disease outcomes that are mediated through individual behaviours and cognitive dispositions. The account is illustrated through analysis of statistical associations of individuals’ educational attainment levels with measures of income, gender, and race serving as control variables.

While there is acknowledgment that mediators between the educational attainment and health outcomes association include income, access to health care and labour market advantages of higher paying jobs, these are explained away as accounting for only a limited proportion of variance. Indeed, great care is devoted to ascertaining that income accounts for only about a third of the educational attainment and health outcomes relationship.

There certainly is no analysis of income and labour market features and how greater equality would serve to weaken education, income and health outcomes relationships. Instead, emphasis is placed on educational attainment as promoting these precursors of health: healthy behaviours, information and cognitive skills, life preferences, and perceived rank in society. The authors note that these characteristics can be considered within the availability of resources as predictors of health as presented in Link and Phelan’s fundamental cause theory.

However, Link and Phelan’s^45^ concept of fundamental causes was primarily concerned with socioeconomic status and its relationship with money, power, prestige and a variety of interpersonal resources such as social support and networks. In fact, the words education, schooling or educational attainment do not appear at all in their classic 1995 paper. Their 2010 paper however does discuss education as a fundamental cause in their presentation of others’ research findings.^46^ It is notable that the 2010 Phelan and Link paper contains policy recommendations that do not emphasize educational attainment as a focus:

The most direct policy implication of the theory is that, if we redistribute resources in the population so as to reduce the degree of resource inequality, inequalities in health should also decrease. Policies relevant to fundamental causes of disease form a major part of the national agenda, whether this involves the minimum wage, housing for homeless and low-income people, capital-gains and estate taxes, parenting leave, social security, head-start programs and college-admission policies, regulation of lending practices, or other initiatives of this type^46, p.S37^

Cutler and Lleras-Muney’s policy recommendations are limited to means of improving educational attainment with no mention of how living and working conditions impact individuals’ educational attainment, much less the economic and political structures that drive these conditions. There is no allusion to the distribution of income and wealth within a society and how these would not only affect educational attainment but also health.

Hummer, R. A., & Hernandez, E. M. (2013). The effect of educational attainment on adult mortality in the United States. *Population Bulletin*, *68*(1), 1-16.

In this paper attention is paid to the relationship between education attainment and life expectancy in the USA. Special attention is paid to these relationships among white, black, and Latino Americans and it is noted that educational attainment-related disparities in life expectancy are increasing.

The authors document the wide variation in mortality rates by educational attainment. These rates for white women without high school completion are almost four times higher than those for white women with 16 or more years of education. White men without high school completion have rates four times higher than those with 16 years or more education.

Interestingly, the authors also draw upon Link and Phelan’s “fundamental cause theory” to explain how educational attainment has an impact on health and life expectancy. Yet, as noted above, Link and Phelan’s 1995 and 2010 papers were primarily concerned with socioeconomic status and money, power, prestige and interpersonal resources, not education. Nonetheless, Hummer et al. argue:

They propose that educational attainment is a root or primary cause of health and longevity because it affects multiple diseases, works through multiple mechanisms to influence health and longevity, serves as a resource that can be used to avoid health risks or lessen the impacts of disease, and continues to influence health and longevity even when the mechanisms linking education to health and longevity change (p. 8).

Hummer and colleagues argue that “socioeconomic attainment—occupational status, income, and wealth accounts for 30 to 40 percent of mortality differences between individuals with the highest and lowest levels of education in the United States:

In particular, increased income improves health-related lifestyles, affords individuals the chance to live in better housing located in safer neighborhoods, and enhances individuals’ access to higher-quality food. Moreover, higher occupational status and income also help U.S. individuals acquire comprehensive health insurance plans, which is important for accessing high quality health care on both routine and emergency bases (p. 9).

Another 30 percent is accounted for by health behaviours such as tobacco use, regular exercise, good nutrition, and preventive health. The remainder is attributed to “cognitive functioning” that allows educated individuals to “learn to evaluate risks and learn to process information and make decisions that enhance health and longevity.”

The authors provide a sense of how they emphasize educational attainment as a social determinant of health in their discussion of *Upstream* versus *Downstream* factors that link educational attainment to life expectancy. The highest upstream issues are subsumed within *Early Life Factors*: Parental Socioeconomic Status, Physical and Mental Health, Peer and Social Context, and Intelligence and Genetics. These lead to different levels of *Education*, that is, Educational Attainment which then produced *Adult Mechanisms* of SES (occupation/Income/Wealth), Social and Psychological Resources, Health-related Behavior and Cognitive Functioning. *Outcomes* are Annual Rate of Mortality and Life Expectancy.

The authors make it clear they believe that increasing educational attainment rather than improving individuals’ circumstances either as parents, children, adolescents, or adults is where policy advocates should be placing attention:

If educational attainment is shown to affect mortality and life expectancy, then increased educational investments can be made. In contrast, a research focus on a multidimensional concept such as socioeconomic status may result in murky policy recommendations: Is it straightforward, for example, to increase individuals’ socioeconomic status? (p. 9).

However, their policy recommendations are vague.

In the case of educational differences in adult mortality, enhanced investments in the early educational progress of American students helps ensure that they will complete high school and perhaps even pursue a higher degree. With at least a high school degree, such individuals may also experience better life-long employment opportunities, have enhanced cognitive skills, and have relationships with others who are more highly educated. These examples represent mechanisms by which individuals can make more informed health-related decisions and potentially live healthier and longer lives (p. 11).

Hahn, R. A., & Truman, B. I. (2015). Education improves public health and promotes health equity. *International Journal of Health Services*, *45*(4), 657-678.

In this article, we see the coordinating scientist of systematic reviews on health equity for the CDC Guide to Community Preventive Services and a consulting editor for infectious diseases for the Journal of Public Health Management and Practice explicitly stating “We propose a broad concept of education **as a personal attribute,** which includes not only subject-matter knowledge, reasoning, and problem-solving skills, but also awareness of one’s own emotions and those of others and control of one’s emotions (i.e., “emotional intelligence”) and associated abilities to interact effectively (emphasis added) (p. 659).

The authors make the argument that not only is education a determinant of health but can be seen as being part of health itself. Educational attainment is seen as just one way of considering health with others being grades received and scores on standardized tests. The authors then provide evidence – limited to statistical analyses – that these indicators are causal factors in individuals’ health outcomes.

Not surprisingly, the authors begin with health risk and protective behaviours as being associated with academic achievement. These include tobacco and alcohol use, physical activity, carrying a weapon, sexual activity, and watching television. They then consider wages and income – themselves predictive of health – as being determined by education. Nothing is said about the state and distribution of wages and wealth and why relationships between these are especially strong in the USA. Much of the article then goes on to document the multiple health outcome measures associated with less education: circulatory diseases, diabetes, liver disease, and psychological symptoms of sadness, hopelessness, and worthlessness and higher mortality rates and lowered life expectancies.

Hahn and Truman provide a model depicting the pathways from educational attainment to health outcomes. The beginning point is educational attainment acting through three major pathways: Psychosocial Environment, Work, and Health Knowledge, Literacy and Behaviours. The Psychosocial Environment path is about establishing a Sense of Control, Social Standing, and Extent of Social Support. Sense of Control leads to work-related factors, health related behaviours, and extent of stress. Social Standing shapes social and economic resources, and extent of stress. Social Support also leads to social and economic resources, health behaviours, amount of stress, and family stability.

The Work pathway acts through Working Conditions such as exposure to hazards, control/demand imbalances and stress; Work-Related Resources such as health insurance, sick leave and benefits; and Income allowing for quality of housing, neighborhood, nutrition and amount of stress. Finally, the Health Knowledge, Literacy, and Behaviours pathway determines nutrition, risk behaviors, and navigating the healthcare system. All of these pathways shape health outcomes.

The remainder of the paper documents the profound differences in all of these outcomes among Americans differing in educational attainment. Policy implications are limited to strengthening education systems and promoting educational achievement. Interestingly, the authors make the argument that just as other determinants of health are the focus of attention such as agriculture, transportation, immigration, and urban design, so also should education be a focus. Remarkably the links between these determinants – and others not mentioned such as income and housing – with educational attainment go unmentioned. The need to address the societal structures and processes that shape these determinants – and educational attainment goes unmentioned.

Zimmerman, E. B., Woolf, S. H., & Haley, A. (2015). Understanding the relationship between education and health: a review of the evidence and an examination of community perspectives (pp. 347-384). In *Population health*: *Behavioral and social science insights*. Agency for Health-care Research and Quality.

Interestingly, the authors’ introductory statement: “Of the various social determinants of health that explain health disparities by geography or demographic characteristics (e.g., age, gender, race-ethnicity), the literature has always pointed prominently to education” (p. 348) neglects mention of income or social class. After documenting the profound health inequalities associated with educational attainment, the authors ask:

What accounts for the growing health advantages that exist among people with higher educational attainment? Is it what they learn in school, such as how to live a healthy lifestyle, or the socioeconomic advantages that come from an education? (p. 348).

Zimmerman and colleagues place their analysis within Kaplan, Everson, & Lynch’s^47^ *Socioeconomic Model* which, by including at its highest levels, *Social and Economic Policies* and *Institutions* suggests attention will be paid to the structures and processes of society that distribute education as well as income. They then proceed to examine the health benefits of education at the “Individual” and “Community” levels before considering “The Larger Social Context and Social Policy.” Six pages are given to the Individual Level, two to the Community Level, and two and a half to The Larger Context.

The content for the Individual Level sections considers a rather large shopping list of individual skills accruing from greater education attainment that contribute to better health. These include: “cognitive skills, problem solving ability, learned effectiveness, and personal control” … “conscientiousness, openness to experience, extraversion, agreeableness, and neuroticism/emotional stability” … “Locus of control, personal efficacy, personal autonomy, self-directedness, mastery, and instrumentalism” … and “cognitive ability, literacy, reaction time, and problem solving” (pp. 5-6). Finally, educational attainment can provide reading and mathematics skills as well as promote science/health literacy.

In the section *Education Increases Economic and Social Resources* it is acknowledged that educational attainment assists in gaining desirable jobs with greater income and job satisfaction as well as health insurance, and worksite health promotion programs and greater occupational safety. Educational attainment therefore reduces economic vulnerability which can “affect health through a cascade effect on the ability to acquire resources that are important to health: food, stable housing, transportation, insurance, and health care” (p. 7). And attention is devoted to the lifestyle and health care mantras: “Individuals with higher incomes have more resources to purchase healthy foods, to afford the time and expenses associated with regular physical activity, to have easy transportation to health care facilities or work locations, and to afford health care expenses” (p. 7).

Regarding social resources, educational attainment is associated with social support provided through social networks making available monetary and psychological support. These networks can also provide information and act to model desirable behaviors. It is suggested that individuals with educational attainment may have greater membership in civic groups and organizations that provide health-promoting social support, influence, and engagement/attachment.

In the shorter section on educational attainment’s impact at the community level, the authors imply that educational attainment can situate people into neighborhoods with characteristics that promote health. These features may be physical layouts, services, sociocultural values and enhanced reputations. They may experience less social disorganization and attain greater social capital and collective efficacy. In addition, such neighborhoods are safer, experience less “crime, unemployment, poverty, and exposure to physical hazards” (p. 358) and provide higher educational quality schools.

Maintaining the well-worn mantras of healthy lifestyles and access to healthcare, those with greater educational attainment are seen as living in neighborhoods with greater access to food outlets with healthy food, spaces and facilities for physical activity, and access to healthcare. These neighborhood characteristics that make them attractive to “business, employers, and investors” (p. 10).

The authors discuss the *Larger Social Context and Social Policy* checking off all the main pronouncements from the World Health Organization and others: “Health inequities are driven, in large part, by the social context in which people are born, live, and work, that is, the social policies that shape resources, institutions, and laws; the economic system through which material and financial resources are created and distributed; and the social norms that govern interactions” (p. 360). They go on to declare: “The World Health Organization calls for improved living and working conditions, social protection policy supportive of all, reduced inequality, and strengthened governance and civil society” (p. 360).

Yet their discussion of “employment and wealth-building opportunities of workers and the marketability of an education” is focused on how technological shifts of “tradable” sectors of technology and finance” increasingly require advanced job skills. Those without educational attainment are therefore “vulnerable to long-term economic hardship.” Rather than consider the forces that make some jobs health sustainable and others not, their concern is focused on how:

Educational opportunities, however, are not equally distributed in the United States. Public school funding, largely dependent on local property taxes, varies widely both within and between states. The best funded school systems in the United States have per pupil expenditures almost four times the per pupil expenditures in the lowest spending schools (p. 361).

Inequalities associated with race, gender, ethnicity, sexual orientation, and disability are recognized as contributing to both educational attainment and income differences yet nothing is said about how to rectify these unjust arrangements. Finally, a whole slew of issues are raised primarily in relation to how they contribute to inequalities in educational attainment: low birth weight, unstable home and community life, and family and neighborhood socioeconomic status. They do so through biological pathways related to neuroanatomy and neuroplasticity, endocrine disruption, immune dysregulation, and epigenetic changes. Education is suggested to be, at least in part, a proxy for these issues.

The gaze of the authors is firmly implanted in the importance of promoting individuals’ educational attainment as a means towards health. The fallacy that raising the educational levels of every American will somehow eliminate low paying employment, insecure housing, unsafe neighborhoods, and food insecurity and the stresses associated with these experiences, thereby improving health, remains the elephant in the room. The need to address the economic and political structures that shape the quality and distribution of the social determinants of health remains unstated.

### Works Focused on Income

Our preceding analysis suggested that while emphasis on income as a social determinant of health would not necessarily direct attention to broader factors, they almost always do. While we expected to see some conceptualizations that emphasized income as influencing behavioural risk factors and access to healthcare, the most striking finding of our analysis is the emphasis placed upon public policy that distributes income and other resources among the population and means of making these distributions more equitable. More recent work is concerned with structures of welfare states and even economic systems themselves and how they distribute income amongst the population. This provides a stark contrast to the educational attainment emphasis with its focus on individual educational attainment. Five documents demonstrate the range of the income approach (see Table 4 presented earlier).

Benzeval, M., & Judge, K. (2001). Income and health: The time dimension. *Social Science & Medicine*, *52*(9), 1371-1390.

Benzeval and colleagues^38^ develop the concepts of income potential and health capital to explicate how social determinants of health shape health: Income potential is the accumulation of abilities, skills and educational experiences in childhood that are important determinants of adult employability and income capacity. Education is the key mediator in this association, being strongly influenced by family circumstances in childhood and a central determinant of an individual’s income in adulthood.

Health capital is the accumulation of health resources, both physical and psychosocial, inherited and acquired during the early stages of life which determine current health and future health potential. In this model, an individual’s circumstances in childhood are a result of their parents’ characteristics, their objective living conditions, and other aspects of their social environments. These all contribute to their immediate health outcomes and their potential to acquire income in adulthood. As adults, both the experiences of childhood and adult situations then go on to influence health as adults.

They concluded that financial circumstances in childhood are important determinants of educational attainment and health capital. Two other important determinants during childhood are parental education and family disruption. Controlling for accumulated health capital and educational attainment, recent financial circumstances and social roles, especially employment are also significantly associated with health outcomes. When controlling for all these factors, childhood circumstances still have a significant effect on health.

They propose that policies to reduce poverty, especially for families with children, should be an essential ingredient in any concerted effort to tackle health inequalities. There should be focus on tackling worklessness among adults, improving wages by raising minimum wages and revising taxation levels. There should also be a focus on education and training and providing improved social security benefits. They conclude

Financial circumstances in childhood are an important determinant of an individual’s educational attainment and health capital as they enter adulthood. These in turn have a significant effect on people’s living standards and health in adulthood, and low incomes then also have a detrimental effect on health (p. 110).

Marmot, M. (2002). The influence of income on health: Views of an epidemiologist. *Health Affairs*, *21(*2), 31-46.

In this widely cited article Marmot provides his view of how income plays a significant causal influence upon health through three different pathways. The first is its direct effect on the material conditions necessary for biological survival; the second the ability to participate in society; and third, the opportunity to control life circumstances. The effect of income on health increases as fewer goods and resources are publicly provided. Indeed, he argues under present USA circumstances, policies for counteracting growing income inequalities through the tax and benefits system and the provision through public policy of a variety of resources appear justified.

Marmot pays particular attention to the relationship of income to infant mortality rates and contrasts the view that health outcomes are a function of ignorance versus the presence of poor living and working conditions. He argues for improving the quality of the social environment by using the tax and benefits system to improve the living standards of those who are worse off. This is particularly important for those at the lower end of the income scale because of the material deprivation, restriction of one’s social participation and opportunity to control exercise control of one’s life. He notes that pretax income inequalities have increased in many countries and the failure to reform the tax and transfer system and invest in public goods will damage health.

Coburn, D. (2004). Beyond the income inequality hypothesis: Class, neo-liberalism, and health inequalities. *Social Science & Medicine*, *58*(1), 41-56.

In a paper expanding upon Wilkinson’s view that income inequality is an important determinant of health, Coburn links income as well as income inequality to the onset of neoliberalism as a governing ideology. Recognizing the literature that income is an important determinant of health, Coburn argues that the distribution of income becomes skewed when there is an emphasis on the marketplace as the arbiter of distribution of resources such as work type, education, health care, housing, transportation, and nutrition.

**Figure 1.**
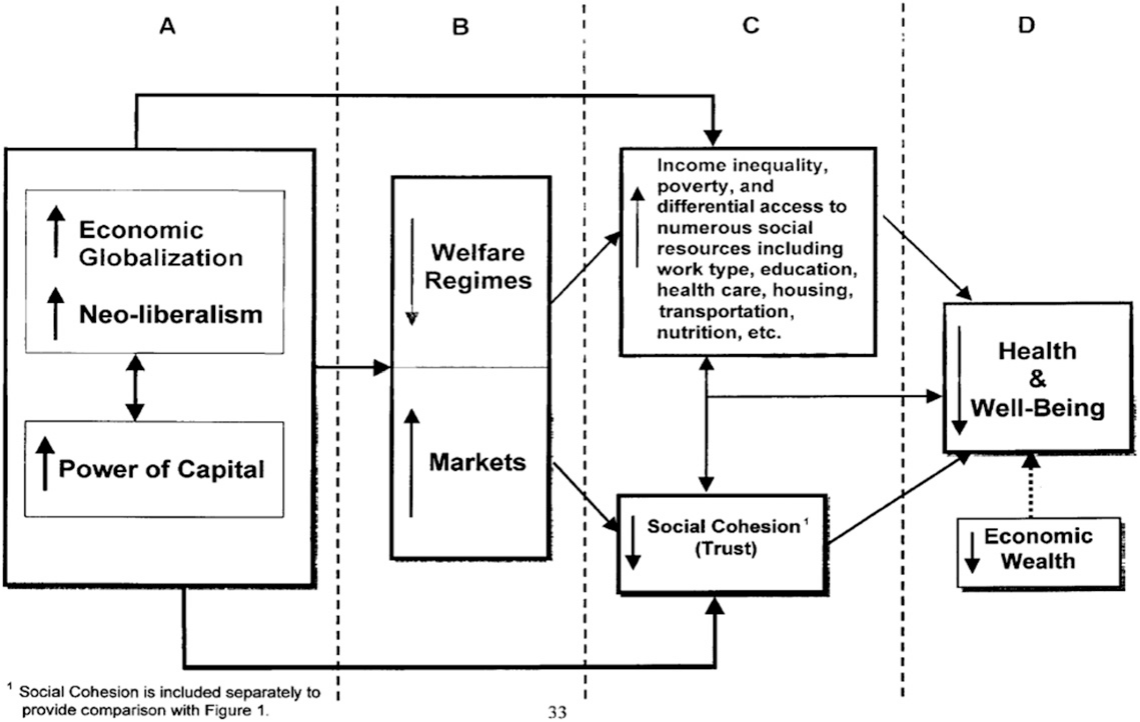
The class/welfare regime model. Source: Coburn, D. (2004). Beyond the income inequality hypothesis: Class, neo-liberalism, and health inequalities. Figure 2, p. 44. *Social Science & Medicine*, *58*(1), 41-56.

Coburn outlines how economic globalization is associated with both neoliberalism and the power of capital (investment monies) to shape public policy that degrades the social determinants of health. These forces interact with a nation’s form of the welfare state – social democratic is best while liberal is worse – to shape how these forces play out in a variety of indicators. The emphasis on societal structures and processes informed by a critical view of how social class plays out in contemporary global capitalism is the key insight of this work. Figure 1 shows the complexity of Coburn’s approach in which income is embedded.

Benzeval, M., Bond, L., Campbell, M., Egan, M. Lorenc, T., Petticrew, M. & Popham. F. (2014). *How does money influence health?* Technical Report. Joseph Roundtree Foundation.

**Figure 2.**
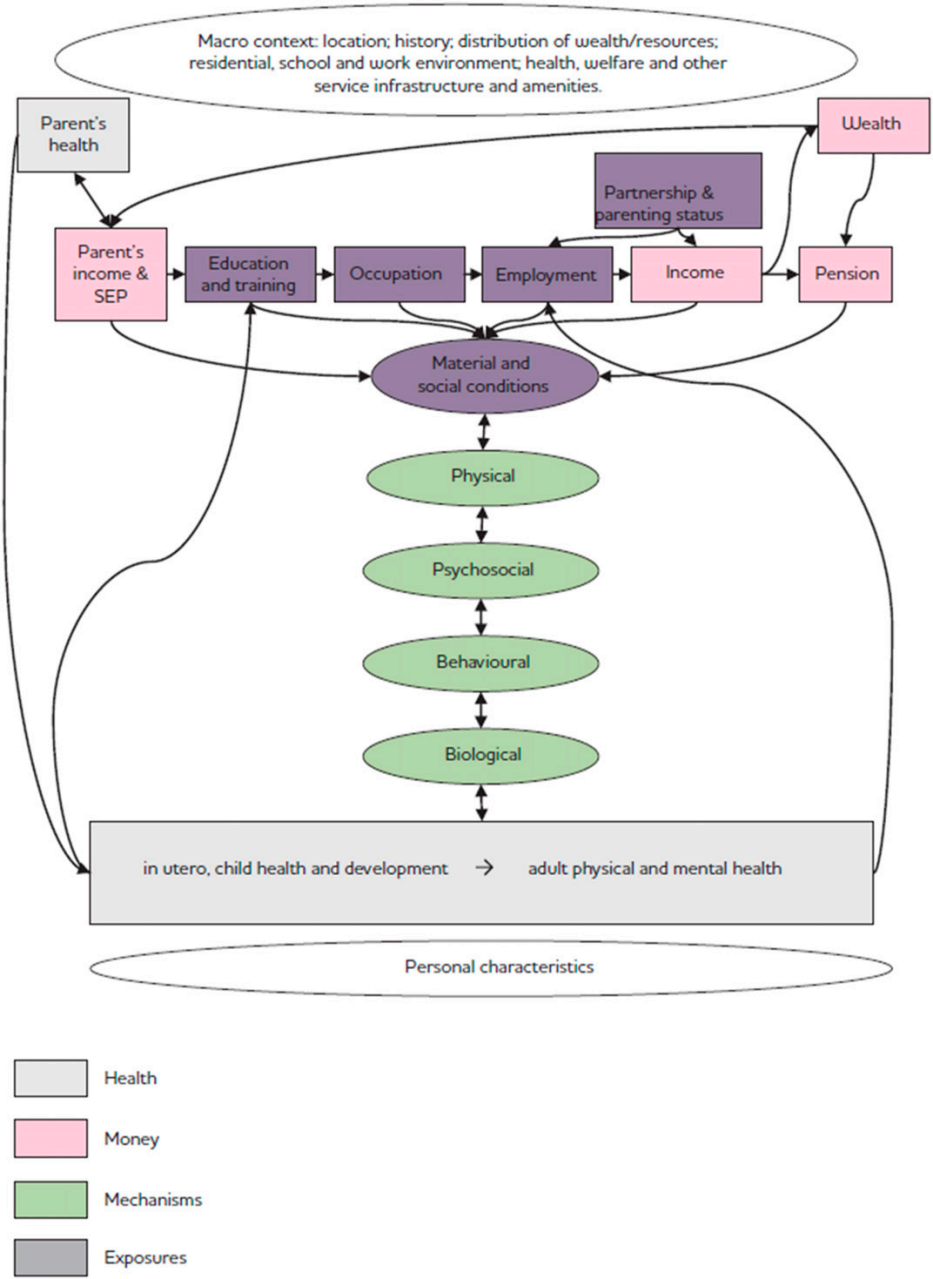
Pathways between income and health. Source: Benzeval, M., Bond, L., Campbell, M., Egan, M. Lorenc, T., Petticrew, M. & Popham. F. (2014). How does money influence health? Figure 4, p. 28. Technical Report. Joseph Roundtree Foundation.

This more recent report brings together what is known about the impact of income on health and what can be done about it. The report outlines materialist, psychosocial, and behavioural pathways between income and health pointing to issues of advantage and disadvantage, psychosocial stress, and adoption of health threatening behaviours as a means of coping with disadvantage. Importantly, the adverse health effects associated with living with low incomes also affects education and employment opportunities. Figure 2 shows how these kinds of analyses direct attention to broader issues well beyond the attributes of individuals.

The report outlines a slew of policy recommendations that flow from this analysis. These include a group termed as welfarist, concerned with levels of benefits and taxation policies to produce a standard of living that allows for health. Authorities should also provide benefits that protect from sudden shocks such as job loss, relationship breakdown and other adverse life events. Importantly, since hardship is not restricted to the unemployed, means of responding to the income and health relationship should include adequate minimum wages and provision of childcare expenses. Another set of policies are about redistributing income through taxation, family benefits, pensions and tax credit systems.

The report recognizes the importance of providing accessible and tailored employment services for the disadvantaged but warns that “jobs that are low paid and/or have poor working conditions can make such strategies appear problematic, as can barriers related to welfare entitlement and competing family demands” (p. 49). Finally, the authors call for “reform of structures, practices and attitudes that discriminate against people with impairments and lead to their increased risk of experiencing poverty” (p. 49).

Berkowitz, S. A. (2022). The logic of policies to address income‐related health inequity: A problem‐oriented approach. *The Milbank Quarterly*, *100*(2), 370.

The author points out that the literature describing the relationship between income and health is definitive as is the finding of a gradient between income and health such that income is related to health outcomes from very low to very high income. The author also notes the striking variety of associations of income with health outcomes and why these relationships are so strong:

This variety includes both categories of diseases with widely varying pathophysiologic mechanisms (e.g., cardiometabolic conditions, malignancies, mental health) and conditions that occur at different times across the life course (e.g., infancy and childhood, early adulthood, older age). Beyond noting these findings, however, it is worth thinking about why the relationship between income and health outcomes, almost any outcome, is so strong (p. 373).

A wide range of policies are presented that could reduce income-related health inequalities. He clearly points to reforms in the structures that are responsible for health inequalities. Importantly he delineates the important role that power plays in shaping these structures. The maldistribution of income is a result of the imbalance of power those among those who create wealth. The policy areas delineated are titled Social Enfranchisement, Social Insurance, Social Assistance, and Social Services. In regards to Social Enfranchisement, the policies that would address these inequalities would focus on policies that tax capital gains income at lower rates than those for labor income as well as others:

A variety of policy approaches can be used to adjust the balance of power that determines how factor income is distributed. One way is to provide a wage floor through minimum wage laws. Another way is to redress power imbalances that may drive down wages. Examples include policies that support unionization, wage boards, and sectoral bargaining; regulate at-will employment; and promote codetermination (including workers’ representation in corporate governance) (p. 380).

**Figure 3.**
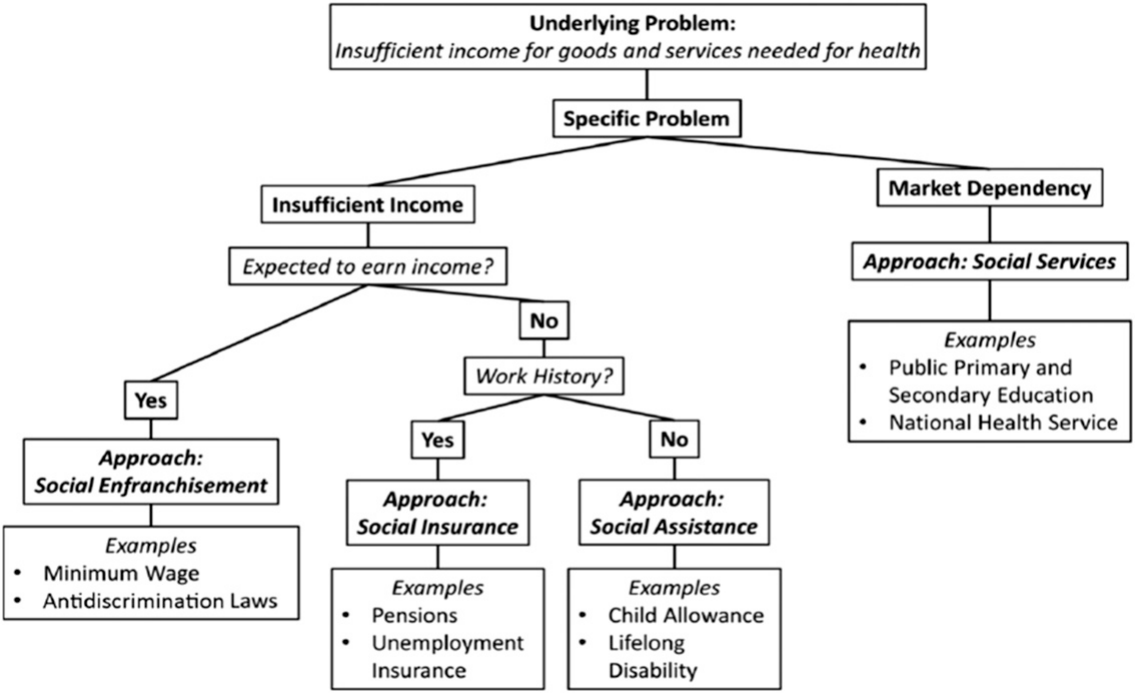
Framework of policy approaches to address income-related health inequities. Source: Berkowitz, S. A. (2022). The logic of policies to address income‐related health inequity: A problem‐oriented approach. Figure 2, p. 378. The Milbank Quarterly, 100 (2), 370.

The author also points to various forms of discrimination such as racism and sexism that unduly skews income and policies to respond to these: “Examples of such policies are the passage and enforcement of anti-discrimination laws related to hiring, promotion, and compensation; affirmative action programs; and the enforcement of rules and regulations regarding workplace hostility that may be directed to individuals on the basis of their race, ethnicity, gender, or other types of ascriptive identity” (pp. 380-381) Figure 3 shows additional areas of concern and policy responses in this multifaceted approach to understanding and addressing income as a social determinant of health.

### Analysis

There is a striking contrast between those emphasizing education versus income as a social determinant of health. When education is considered, the analysis is individualized, uncritical of existing societal structures and processes, and clearly accepting of the status quo. In contrast, when income is considered, the analysis quickly moves to consider the structures and processes that distribute income and other resources amongst the population.

Certainly, there is no reason that education proponents cannot be concerned with how features of economic and political systems inequitably distribute educational opportunities and propose redistributive educational and income remedies. But their views of the nature of the health inequalities problem and its solution usually make this difficult, situated as they are within liberal/reformist paradigms of how societies work. And there is certainly no reason that income proponents cannot provide individualistic solutions for deficiencies in income such as psychological counseling and personal and workplace strategies for stress reduction, but again, they usually do not, instead usually directing their attention to the structures and processes of society that inequitably distribute income.

It would not be surprising to find that the education approach is more accepted within societies where the influence of the corporate sector (i.e., in liberal welfare states such as Canada, the UK, and USA) is greater as it says nothing to question their power and influence on public policy that distributes the resources necessary for health. We illustrate how this plays out through a critical examination of the Pathways to Education project, which attempts to address educational disadvantage in Canada.

### On the Ground: The Canadian Pathways to Education Program as Exemplar

We chose the Canadian Pathways to Education project as an illustration of how the education approach play out as it is the most prominent program in Canada addressing the educational needs of disadvantaged youth. It also illustrates how corporate domination of its Board of Directors may reinforce the liberal/individualist approach to understanding social and health inequalities and responding to such inequalities.

The Pathways program was first implemented in the Regent Park community in Toronto in 2001.^48,49^ In 2005 the charity Pathways Canada was established to fundraise and expand the program across Canada. It grew to three more sites in Toronto and sites in Halifax, Hamilton, Kingston, Kitchener, Montreal, Ottawa, Shawinigan, Sherbrooke, Mashteuiatsh, and Winnipeg. The program targets disadvantaged youth to reduce high school dropout rates and increase access to enrolment to post-secondary institutions.

Students enrolled in the Pathways program receive social, academic and counseling supports such as an adult role model to guide students’ decisions, mentors in fields the students are interested in, financial support (lunch vouchers, transit passes, scholarships), and life planning counseling after they complete high school.^6^

Overall Pathways appears to be a successful model for combating low graduation rates in at-risk communities. High school completion rates have risen from 44% to 59% and postsecondary enrolment rates from 38% to 57% in Regent Park. Pathways also boasts that across its programs income of pathways graduates increased by 19%, employment rates by 14%, with less reliance on social assistance.^48,49^ For the most recent year, the graduation rate was 79%.

The program has expanded since beginning with 115 students in 2001-2002 to hosting 7962 in 2022-2023 in 31 Pathway Programs across Canada.^6^ Its revenues were $38,261,736 coming primarily from governments (61%), corporations (10%), Foundations and Agencies (23%) and individuals (6%).^50^ While Pathways reports increasing the annual income of their graduates by 19% helping them avoid poverty, it says nothing about why so many of their clients live on low incomes and are in need of such a program. Its unwillingness to take positions on the factors that make such programs necessary are problematic and consistent with our analysis of the limitations of the educational attainment approach to promoting equity. This unwillingness to address broader issues may be reinforced by the composition of its national Board of Directors.

### What Pathways to Education Neglects

While Pathways to Education is focused on supporting disadvantaged youth in their educational activities, it could use its profile to identify the important factors that create the need for such programs. Assisting close to **8000** students a year is commendable, yet Campaign 2000 reports that in 2022 Statistics Canada identified **1.4 million children** as living in poverty across Canada of which 360,000 fell into poverty over the last 2 years.^51^ The total represents 18.1% of all children in Canada.

Low income is the most important driver of low educational attainment in Canada and is caused by low wages and social assistance and other benefit levels, lack of unionization of the workplace, and racism and discrimination that concentrates poverty in particular groups such as youth of colour, Indigenous peoples, and those living with disabilities.^52^ All of these are embedded within what Lynch^53^ identifies as three governance taboos that make reducing social and health inequalities difficult, if not impossible: redistribution, social spending, and market regulation. It is beyond the scope of this article to examine these taboos in detail, but these are strongly reinforced by corporate sector lobbying that has blocked such activities.^54^

We carefully reviewed all Pathways to Education reports, documents and website content to find statements about low income, its causes, and what can be done to address its role in fostering lower educational attainment that makes the Pathways program necessary. There is no such content in these documents and no evidence that the program has carried out any form of advocacy to have governing authorities address these issues.

One of the reasons why any form of advocacy to address these societal drivers of educational disadvantage is missing from Pathways activities may be its domination by the corporate sector which has a well-established opposition to forms of redistribution, social spending, and market regulation.^54^ Azadian et al.^55^ and Raphael et al.^56^ provide an analysis of how such corporate domination of disease associations and food banks and food diversion projects in Canada is associated with these agencies' lack of advocacy for structural changes in society that would reduce chronic disease and food insecurity, an analysis clearly applicable to the Pathways to Education absence of public policy advocacy to reduce the material disadvantage that drives the lack of educational attainment.

### Pathways Board of Directors

The Board has responsibilities of directing Pathways activities: “As the governing body of Pathways to Education Canada, the National Board of Directors oversees our policy development, sets our strategic direction, and monitors our performance against our goals.”^57^ As shown in Table 5, The Board Executive is made up completely of high-level corporate executives. Corporate executives also constitute a majority of the other Directors. It is not surprising that Pathways takes no forays into the public policy arena as the corporate sector actively engages in public policy advocacy to limit redistribution, unionization, and social spending.^32^

**Table 5.** National Board of Directors, Pathways to Education.

Status	Name	Employer	Description	Position
Chair	Vincent Mercier	Davies LLP	Law Firm focused on Business Law	Partner
Vice-Chair	Lori Pearson	Brookfield Asset Management	Global Investment Firm	Sr. Managing Partner & COO,
Treasurer	Katherine Gibson	Royal Bank of Canada	Major Chartered Bank	SVP Finance & Controller
Member	David Ain	Egon Zehnder	Business Consultant	Managing Partner
Member	Darren Googoo	Membertou First Nation	Native Band	Director of Education
Member	Stacey Grant-Thompson	ManagingLife	Insurance Company	Strategic Advisor
Member	Wes Hall	Kingsdale Advisors	Strategic Shareholder Advisory Firm	Executive Chairman & Founder
Member	Myriam Legault			Educator
Member	Fabrice Morin	Canada Life	Major Insurance Company	EVP, Canadian Operations
Member	Pamela Sugiman	Toronto Metropolitan University	Major University	Dean, Faculty of Arts
Member	Nisita Tappata	Integrations Ecosystem, POAP Inc.	Organizational Consultants	Head of Integration

Source: Pathways to Education.^57^ Our leadership. https://www.pathwaystoeducation.ca/our-leadership/.

## Conclusion

Our analysis suggests that the eventual solution of inequalities in health is likely best served by taking a materialist/structuralist approach towards understanding and responding to these inequalities. A materialist/structuralist approach directs attention to the value of addressing both educational attainment and income inequalities through analysis and reform or transformation of the societal structures that distribute them. In practice however, these recommendations for reforms or transformations are usually associated with the income as a social determinant of health camp. Current approaches towards education as a social determinant – with their emphasis upon individual attributes and neglect of societal structures and processes -- are unlikely to promote health equity through the reduction of health inequalities.

In this article we have provided what we believe is a needed analysis of the implications of taking an educational attainment versus an income lens to understanding social and health inequalities. We believe there are important implications of these differing approaches. An educational attainment approach fits into a liberal/individualist frame – usually utilizing positivist methodologies focused on individual attributes and is consistent with Moral Underclass and Social Integrationist notions of social exclusion and potentially leading to stigmatization and victim blaming.^58^ More importantly it ratifies the status quo by providing existing economic and political systems with a stamp of approval.

And clearly the status quo in Canada and elsewhere is beginning increasingly problematic with a polycrisis of growing income inequality, precarious work, unaffordable housing, increasing food insecurity, and the climate crisis, amongst others.^59^ There is no necessity that this be the case. We can certainly expand existing programs to promote educational attainment but do so with advocacy for the structural changes that make such programs necessary. This is where the analyses provided by the income perspective can be helpful. The limitations of the educational attainment need to be made explicit and the subject of debate and further analysis.

Regarding the Pathways to Education and other similar programs adopting an advocacy approach to broader issues, there may be pushback to such a shift from the powers-that-be that benefit from the current inequitable distribution of income, influence, and power. But such advocacy is clearly necessary for improving the situations of the disadvantaged in Canada – including their greater risk of lower educational attainment. Considering that only a small proportion of Pathways’s funding comes from the corporate sector, a good first step would be diversifying the members of its Board of Directors thereby removing one of the strongest barriers to advocacy for public policies of redistribution, social spending and market regulation that would lead to a more equitable and just society. The next step would be working with a wide range of civil society organizations concerned with poverty reduction and reducing discrimination, thereby strengthening the voice of those disadvantaged by current societal structures and processes and making reform or transformation of these structures that create disadvantage more likely.

## Notes

1. Wealth is certainly as important or more so as a social determinant of health than income. However, income has been the subject of much more research and public policy attention – see especially the work of Wilkinson and Pickett.^60^ In addition, the implications that flow from an income focus – redistributing income through progressive income and other tax structures, raising wages, improving benefit programs through increased social spending, and managing the economy by ensuring employment security through legislation and promoting unionization and collective agreement bargaining may be more immediately achievable than redistributing wealth.^53^

2. Veenstra and Vanzella-Yang^61^ considers both educational attainment and income as determinants of health but only in regard to carrying out research. Their paper does not provides policy recommendations.
